# Characterization and Dynamics of Intracellular Gene Transfer in Plastid Genomes of *Viola* (Violaceae) and Order Malpighiales

**DOI:** 10.3389/fpls.2021.678580

**Published:** 2021-08-27

**Authors:** JiYoung Yang, Seongjun Park, Hee-Young Gil, Jae-Hong Pak, Seung-Chul Kim

**Affiliations:** ^1^Research Institute for Dok-do and Ulleung-do Island, Kyungpook National University, Daegu, South Korea; ^2^Institute of Natural Science, Yeungnam University, Gyeongsan, South Korea; ^3^DMZ Botanic Garden, Korea National Arboretum, Yanggu, South Korea; ^4^Department of Biology, School of Life Science, BK21 FOUR KNU Creative BioResearch Group, Kyungpook National University, Daegu, South Korea; ^5^Department of Integrative Natural Sciences for the East Sea Rim, Kyungpook National University, Daegu, South Korea; ^6^Department of Biological Sciences, Sungkyunkwan University, Suwon, South Korea

**Keywords:** intracellular gene transfer, *Viola*, *infA*, *rpl32*, *rps16*, Malpighiales

## Abstract

Functional gene transfer from organelles to the nucleus, known as intracellular gene transfer (IGT), is an ongoing process in flowering plants. The complete plastid genomes (plastomes) of two Ulleung island endemic violets, *Viola ulleungdoensis* and *V. woosanensis*, were characterized, revealing a lack of the plastid-encoded *infA*, *rpl32*, and *rps16* genes. In addition, functional replacement of the three plastid-encoded genes in the nucleus was confirmed within the genus *Viola* and the order Malpighiales. Three strategies for the acquisition of a novel transit peptide for successful IGT were identified in the genus *Viola*. Nuclear *INFA* acquired a novel transit peptide with very low identity between these proteins, whereas the nuclear *RPL32* gene acquired an existing transit peptide *via* fusion with the nuclear-encoded plastid-targeted *SOD* gene (Cu-Zn superoxide dismutase superfamily) as one exon, and translated both proteins in the cytosol using alternative mRNA splicing. Nuclear *RPS16* contains an internal transit peptide without an N-terminal extension. Gene loss or pseudogenization of the plastid-borne *rpl32* and *rps16* loci was inferred to occur in the common ancestor of the genus *Viola* based on an infrageneric phylogenetic framework in Korea. Although *infA* was lost in the common ancestor of the order Malpighiales, the *rpl32* and *rps16* genes were lost multiple times independently within the order. Our current study sheds additional light on plastid genome composition and IGT mechanisms in the violet genus and in the order Malpighiales.

## Introduction

Plastids are important cellular organelles that contain their own genomes and originate from cyanobacteria-like prokaryotes by endosymbiosis (Keeling, 2010). The plastid genome (plastome) undergoes reduction as a consequence of gene loss or endosymbiotic gene transfer (EGT) to the host cell nucleus (Timmis et al., 2004). Many transferred gene products need to be targeted back to the plastids, which involves repair of double-strand breaks by non-homologous end-joining of DNA released from organelles (Kleine et al., 2009). After EGT to the host nucleus, most angiosperm plastomes retain genes encoding 79 proteins, 30 tRNAs, and four rRNAs (Ruhlman and Jansen, 2014). Although the gene content is conserved in angiosperms, plastid-encoded gene loss (e.g., *accD*, *clpP*, *infA*, *ndhA-K*, *rpl20*, *rpl22*, *rpl23*, *rpl32*, *rpoA*, *rps7*, *rps11*, *rps12*, *rps15*, *rps16*, and *rps18*) has been documented across several lineages (Jansen et al., 2007; Park et al., 2018). These gene losses are often the result of functional gene transfer from plastids to the nucleus and are known to occur frequently and represent ongoing processes (Timmis et al., 2004). To conduct this unique function, the transferred plastid genes should acquire nuclear expression elements with transit peptides for the import of products into plastids (Bock and Timmis, 2008). However, the majority of functional transfers in angiosperms are restricted to nine of the 25 protein-encoding genes including acetyl-CoA carboxylase subunit β (*accD*), translation initiation factor A (*infA*), and four large and three small subunit ribosomal proteins (*rpl20*, *rpl22*, *rpl23*, *rpl32*, *rps7*, *rps15*, and *rps16*, respectively).

A number of possible mechanisms involved in functional gene replacement have been proposed, including direct intracellular gene transfer (IGT) to the nucleus, or gene substitution by a nuclear homolog. For example, IGT from plastids to the nucleus in angiosperms has been documented for five plastid genes: *accD* in *Trifolium* (Magee et al., 2010; Sabir et al., 2014), *infA* in Ranunculaceae (Park et al., 2015; Park and Park, 2020), *rpl22* in Fabaceae and Fagaceae (Gantt et al., 1991; Jansen et al., 2011), *Passiflora* (Shrestha et al., 2020), *rpl32* in Ranunculaceae (Park et al., 2015, 2020; Park and Park, 2020), Rhizophoraceae, and Salicaceae (Cusack and Wolfe, 2007; Ueda et al., 2007), *rps7* in *Passiflora* (Shrestha et al., 2020), and *rps15* in *Papaver* (Park et al., 2018). Among these, the transferred plastid *accD*, *rpl32* (Rhizophoraceae and Salicaceae), and *rps7* genes have adopted a transit peptide from an existing nuclear gene, whereas the *infA*, *rpl22*, *rpl32* (Ranunculaceae), and *rps15* genes acquired a novel transit peptide. Gene substitution for plastids in the nucleus has been documented for three genes: *accD* in Brassicaceae (Schulte et al., 1997; Babiychuk et al., 2011), *Geranium* (Park et al., 2017), and Poaceae (Konishi et al., 1996; Gornicki et al., 1997), *rpl20* in *Passiflora* (Shrestha et al., 2020), *rpl23* in spinach (Bubunenko et al., 1994), *Geranium* (Weng et al., 2016), *rps16* in *Lupinus* (Keller et al., 2017), *Medicago* and *Populus* (Ueda et al., 2008), *Passiflora* (Shrestha et al., 2020), and Ranunculaceae (Park et al., 2020; Park and Park, 2020). The *accD* and *rpl23* loci were substituted by a cytosolic homolog of eukaryotic origin, whereas *rps16* and *rpl20* have been substituted by cytosolic homologs of mitochondrial origin (i.e., the nuclear genome had already acquired mitochondrial *rps16* and *rpl20 via* IGT).

The cosmopolitan genus *Viola* L. (Violaceae, Malpighiales) consists of approximately 600 species and is distributed mainly in temperate regions and high mountains in tropical regions (Yoo and Jang, 2010; Marcussen et al., 2014). *Viola* presumably originated in South America and subsequently dispersed into the Northern Hemisphere approximately 18 million years ago (Early Eocene), and allopolyploidy accounts for a significant portion of the speciation events within the genus (67–88%; Ballard et al., 1998; Marcussen et al., 2012, 2014). Several important characteristics, such as stigma morphology, pistil shape, petal color, chromosome number, and geographical distribution, in conjunction with several conventional molecular markers, have provided certain baseline classification systems and revealed important evolutionary processes. Nevertheless, we are still far from fully understanding the complex interspecific relationships involving *Viola* (Gingins de la Sarraz, 1823; Becker, 1925; Clausen, 1964; Ballard, 1996; Ballard et al., 1998; Marcussen et al., 2014). In particular, a high proportion of allopolyploidy within the genus, with numerous hybrid and polyploid complexes, in conjunction with highly variable morphological traits, has hampered violet systematists sufficiently to fully reveal complex reticulating events among the species. Korean *Viola* represents one of the most important angiosperm lineages, with 43–64 recognized taxa (Yoo and Jang, 2010), but little is known regarding the contribution of allopolyploidy in the speciation of phylogenetically and taxonomically complex groups. Of the numerous hybrid and polyploidy complexes, *Viola woosanensis*, endemic to Ulleung Island in Korea, has been suggested to be of hybrid origin involving other Ulleung Island endemic *V. ulleungdoensis* and East Asia-restricted *V. chaerophylloides* (Gil and Kim, 2016). As we assembled the plastomes of these *Viola* species to gain insights into the origin and evolution of Ulleung Island endemics, we discovered the absence of *infA*, *rpl32*, and *rps16*. Although plastomes of other congeneric species of *Viola* (i.e., *V. mirabilis*, *V. radeana*, *V. phalacrocarpa*, *V. japonica*, *V. seoulensis*, and *V. websteri*) have been characterized in Korea, the presence/absence of these genes has not been explored (Cheon et al., 2017, 2019, 2020). However, Menezes et al. (2018), in comparative plastid genome analysis among species in the order Malpighiales, reported the absence of *rps16* and *rpl32* in a single species of Violaceae (*V. seoulensis*) and in Salicaceae (*Populus alba* and *Salix purpurea*). This raises the question of how frequent the absence of *rps16* and *rpl32* genes is in the *Viola* genus and whether the absence of these genes in plastid genomes was transferred to another genome compartment or lost completely.

In this study, we assembled two plastome sequences of the violet species *V. ulleungdoensis* and *V. woosanensis* endemic to Ulleung Island, Korea, as genomic resources of insular endemics. We also documented the functional transfer of three genes, *infA*, *rpl32*, and *rps16*, from the plastid to the nucleus *via* IGT or gene substitution. Finally, we determined gene loss and functional transfer to the nucleus within the order Malpighiales and the loss of two genes (*rps16* and *rpl32*) within the phylogenetic framework of the genus *Viola* in Korea.

## Materials and Methods

### Plastid Genome Sequencing, Assembly, and Annotation

Fresh *V. ulleungdoensis* and *V. woosanensis* leaves from single individuals were collected from natural populations on Ulleung Island, off the east coast of the Korean Peninsula. Total DNA was isolated using the DNeasy Plant Mini Kit (Qiagen, Carlsbad, CA, United States), and the paired-end (PE) reads were generated using the Illumina HiSeq 4000 platform (Illumina, Inc., San Diego, CA, United States) at Macrogen Corporation (Seoul, South Korea). The PE reads were assembled *de novo* using Velvet v.1.2.10 (Zerbino and Birney, 2008) with multiple *k*-mers (99–143). From each assembly, the full length of the plastome contig, including only one copy of inverted repeat (IR), was generated. Gene annotation was conducted using Geneious R10 v.10.2.6^1^ using the model plant tobacco plastome (NC_001879) as a reference, and tRNAs were confirmed using tRNAscan-SE v.2.0 (Chan and Lowe, 2019). The annotated plastome sequences have been deposited in GenBank with accession numbers MK228834 (*V. ulleungdoensis*) and MK228835 (*V. woosanensis*). Plastome maps were drawn using OGDRAW v1.3.1 (Greiner et al., 2019).^2^

### Identification of Functional Gene Transfer to the Nucleus in *Viola*

To identify IGT events, the transcriptome from *V. acuminata* (SRR5320546) was assembled *de novo* using Trinity v.2.5.1 (Grabherr et al., 2011). Candidates for nuclear-encoded plastid genes were identified by performing “BlastN” searches using BLAST+ v.2.6.0 (Camacho et al., 2009) against the transcriptomes of *rpl32* and *rps16* gene sequences from *Licania sprucei* plastome (NC_024065), and *infA* gene sequences from *Amborella trichopoda* plastome (NC_005086). In the case of *rps16*, the “BlastN” search was additionally performed using the *Medicago truncatula* (AB365526) nuclear-encoded plastid *rps16* sequences. The NCBI Conserved Domain Database (CDD; Marchler-Bauer et al., 2010) and TargetP v.1.1 (Emanuelsson et al., 2007) were used for functional domain annotation and prediction of transit peptides, respectively.

To confirm the nuclear-encoded plastid gene sequences, polymerase chain reaction (PCR) was performed using total genomic DNA from *V. ulleungdoensis*. Primer pairs were designed based on sequences of the nuclear-encoded *INFA*, *SODcp-RPL32*, and *RPS16* transcripts from *V. acuminata* using Primer3 in Geneious R10 (Supplementary Table 1). The PCR products were purified using InClone™ gels and PCR purification kits (InClone Biotech Co., Seoul, Korea). Sequencing reactions were performed using purified PCR products using BigDye Terminator v.3.1 Cycle Sequencing Kit reagents (Applied Biosystems, Foster City, CA, United States) at GenoTech Corp. (Daejeon, Korea). Genomic sequences and transcripts were aligned using MUSCLE (Edgar, 2004) in Geneious R10.

In addition, seven *Viola* taxa were included: *V. betonicifolia* (SRR13316929), *V. canadensis* (ERR2040403), *V. mandshurica* (SRR5320533), *V. orientalis* (SRR5322130), *V. pubescens* (SRR5353310), *V. tricolor* (ERR2040402), and *V. uliginosa* (SRR1576988). Transcriptome assemblies, BlastN searches, identification of transit peptide and conserved domain, and alignments were performed as described above. Maximum likelihood (ML) analysis was performed using IQ-TREE v.1.6.8 (Nguyen et al., 2014), employing 1,000 bootstrap replications.

### Identification of Functional Gene Transfer to the Nucleus in Order Malpighiales

To better understand the evolutionary fate of the plastid-encoded *infA*, *rpl32*, and *rps16* genes, available Malpighiales transcriptomes from the 1KP project database^3^ and the genomics portal Phytozome v.12.1.6^4^ were searched. Nuclear transcripts were identified by “BlastN” searches (*e*-value cutoff of 1e-6), employing the nuclear-encoded *INFA*, *SODcp-RPL32*, and *RPS16* sequences from *V. acuminata* as query sequences. Phylogenetic analysis was performed to detect gene loss or apparent pseudogenes within the order Malpighiales using 19 plastomes from nine major families (Salicaceae, Passifloraceae, Violaceae, Euphorbiaceae, Malpighaceae, Erythroxylaceae, Clusiaceae, Linaceae, and Chrysobalanaceae; Supplementary Table 2). *Euonymus japonicus* (NC_028067) was selected as an outgroup based on a previous study (Choi and Park, 2016). The datasets were aligned with MAFFT (Katoh and Standley, 2013) in Geneious R10. ML analysis was performed using IQ-TREE v.1.6.8, employing 1,000 bootstrap replications.

### Survey for Loss of Two Plastid-Encoded *rpl32* and *rps16* Genes in *Viola*

To document the presence/absence of two ribosomal protein genes, 19 species of *Viola* were sampled from major lineages of the genus in Korea including sections of *Dischidium*, *Chamaemelanium*, and *Nomimium* (Yoo and Jang, 2010; Supplementary Table 3). Total genomic DNA was isolated from silica gel-dried leaves using a DNeasy Plant Mini Kit (Qiagen, Carlsbad, CA, United States). To detect the *rpl32* gene, the intergenic spacer (IGS) region between *ndhF* and *trnL-UAG* genes was amplified using newly designed PCR primers (Supplementary Table 1). In addition, to detect the *rps16* gene, the IGS region between the *trnQ-UUG* and *trnK-UUU* genes was amplified using newly designed primers (Supplementary Table 1). PCR product purification and DNA sequencing were performed as described previously. Phylogenetic analysis of 19 species of *Viola* based on four chloroplast noncoding regions (three IGS sequences, *atpF-H*, *psbA-trnH*, and *trnL-F*, and one intronic sequence, *rpl16*) was performed to detect gene loss or apparent pseudogenes of *rpl32* and *rps16*. *Hybanthus concolor* was used as an outgroup (Yoo and Jang, 2010; Supplementary Table 3). Sequences were aligned using MUSCLE in Geneious R10. ML analysis was performed with IQ-TREE v.1.6.8 with 1,000 bootstrap replications.

## Results

### General Features of Two Insular *Viola* Plastome Sequences

**Figure 1:**
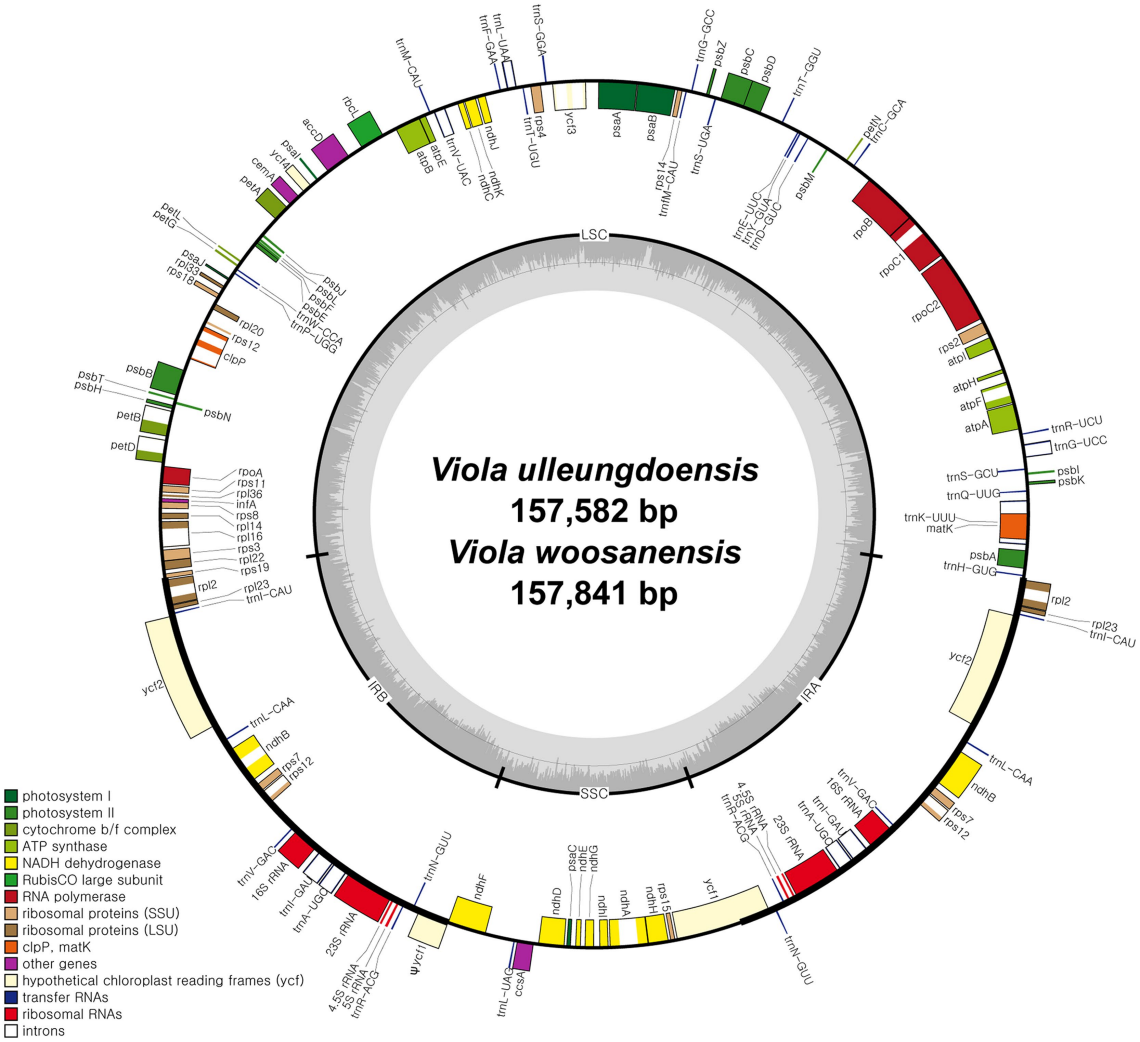
Complete plastome maps of *Viola ulleungdoensis* and *Viola woosanensis*. Genes located outside the circle are transcribed clockwise, whereas those located inside are transcribed counter clockwise. The gray bar area in the inner circle denotes the guanine-cytosine (GC) content of the genome, whereas the lighter gray area indicates the adenosine-thymine (AT) content of the genome. Large single copy, small single copy, and inverted repeat are indicated by LSC, SSC, and IR, respectively. Ψ indicates pseudogenes.

DNA sequencing generated approximately 4.2 and 3.9 Gb of paired-end reads for *V. ulleungdoensis* and *V. woosanensis*, respectively. The data were used to assemble plastomes with a deep coverage of 1,323× for *V. ulleungdoensis* and 511× for *V. woosanensis*. The *V. ulleungdoensis* and *V. woosanensis* plastomes were 157,582 and 157,841 bp, respectively, with a pair of inverted repeats (IR_A_ and IR_B_) of 27,084 and 27,121 bp separated by small and large single copy (SSC and LSC) regions of 17,250 and 86,164, 17,259, and 86,340 bp, respectively (Figure 1 and Table 1). The two *Viola* plastomes encoded 110 genes including 76 protein-coding genes, 30 tRNA genes, and four rRNA genes. The plastid-encoded *infA*, *rpl32*, and *rps16* genes appeared to have been lost in from their original plastomes. Mauve alignment (Darling et al., 2004) of seven Violaceae plastomes showed high structural and sequence similarities (Supplementary Figure 1). In addition, comparative analysis of seven *Viola* plastomes confirmed the loss of three plastid-encoded *infA*, *rpl32*, and *rps16* genes shared by the *Viola* species examined in Korea (Supplementary Figure 1).

**Table 1:** Comparison of *Viola ulleungdoensis* and *Viola woosanensis* plastomes.

	*Viola ulleungdoensis*	*Viola woosanensis*
Size (bp; GC content %)	157,582 (36.3%)	157,841 (36.3%)
LSC length (bp; GC content %)	86,164 (33.9%)	86,340 (33.8%)
SSC length (bp; GC content %)	17,250 (29.8%)	17,259 (30.8%)
IR length (bp; GC content %)	27,084 (42.2%)	27,121 (42.2%)
Number of genes	131	131
Protein-coding genes (duplicated in IR)	76 (9)	76 (9)
tRNA genes (duplicated in IR)	30 (7)	30 (7)
rRNA genes (duplicated in IR)	4 (4)	4 (4)

LSC, large single copy region; IR, inverted repeat; SSC, small single-copy region.

### Functional Replacement of Three Protein-Coding Genes From Plastids to the Nucleus in *Viola*

Our results revealed that the plastid-encoded *infA*, *rpl32*, and *rps16* loci were lost in the assembled *Viola* plastomes. To investigate potential functional transfer to the nucleus, we performed BLAST searches against the *V. acuminata* transcriptome data using plastid gene sequences. A transcript with high sequence identity to *infA* was present, which has a transit peptide (TargetP: chloroplast = 0.542) and a conserved *infA* domain (Table 2 and Supplementary Figure 2). In addition, we also found an open reading frame (ORF) containing the conserved ribosomal protein L32 domain, which included extended sequences of 645 bp downstream from the conserved domain. The first 65 amino acids of the ORF were predicted by TargetP to be a transit peptide (chloroplast = 0.942), which is targeted to the plastid (Table 2). CDD analysis predicted the conserved domain of “Cu-Zn Superoxide Dismutase superfamily” involving the transit peptide and the conserved domain of “ribosomal protein L32” (Supplementary Figure 3A). To further investigate potential alternative mRNA splicing of the *SODcp-RPL32* gene, we queried the *V. acuminata* transcriptome with the ORF. The complete sequence of plastid-targeted *SOD* was identified in the transcriptome (Supplementary Figure 3B). Although the *rps16*-like transcripts were not detected by a “BlastN” search against the plastid-encoded *rps16* gene of *L. sprucei*, two different transcripts were detected using the query sequence of the nuclear-encoded *RPS16* (*M. truncatula*, AB365526). Both transcripts also contained a transit peptide (TargetP: mitochondria = 0.790) and the conserved ribosomal protein S16 domain (Table 2 and Supplementary Figure 4).

**Table 2:** Transit peptide prediction scores of putative nuclear-encoded plastid genes.

	TargetP			
	cTP	mTP	RC	Tplen
*INFA*	**0.542**	0.185	5	21
*RPL32*	**0.942**	0.138	1	65
*RPS16*	0.333	**0.790**	2	7

cTP, chloroplast transit peptide; mTP, a mitochondrial targeting peptide. RC indicates reliability class, from 1 to 5, where 1 indicates the strongest prediction. Tplen means predicted presequence length (cleavage sites). Bold font indicates prediction of localization (chloroplast or mitochondrion).

**Figure 2:**
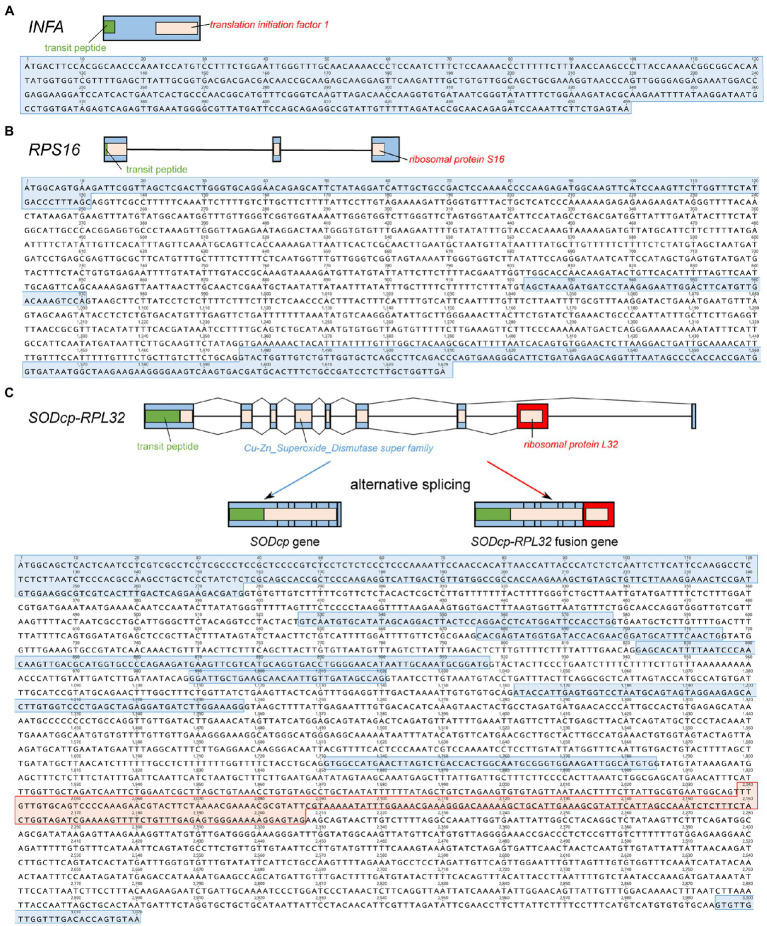
Organization of the nuclear-encoded *INFA*, *SODcp-RPL32*, and *RPS16* genes from *Viola ulleungdoensis*. **(A)** Schematic diagram and nucleotide sequence of the nuclear-encoded *INFA* gene. **(B)** Schematic diagram and nucleotide sequence of the nuclear-encoded *RPS16.* **(C)** Schematic diagram and nucleotide sequence of nuclear-encoded *SODcp-RPL32* as a chimeric gene with alternative splicing. Blue and red boxes denote exons and *RPL32* gene regions, respectively. Green boxes indicate transit peptides.

*Viola ulleungdoensis* was surveyed to identify the nuclear-encoded *INFA*, *SODcp-RPL32*, and *RPS16* loci (for plastids) using PCR and Sanger DNA sequencing. The gene sequence sizes for the nuclear-encoded *INFA*, *RPS16* and *SODcp-RPL32* genes were 459, 1,629, and 3,020 bp, respectively (Figure 2 and Supplementary Table 4). Alignment of the DNA and transcript nucleotide sequences confirmed that the nuclear-encoded *INFA* was an intronless gene, whereas the nuclear-encoded *SODcp-RPL32* and *RPS16* contained eight and two introns, respectively (Figure 2). The conserved domain of nuclear-encoded *RPS16* was included in three exons (Figure 2). In the case of the nuclear-encoded *SODcp-RPL32*, the conserved domain of “Cu-Zn Superoxide Dismutase superfamily” was composed of the first to seventh exon, and the conserved domain of “ribosomal protein L32” was included in the eighth exon (Figure 2).

**Figure 3:**
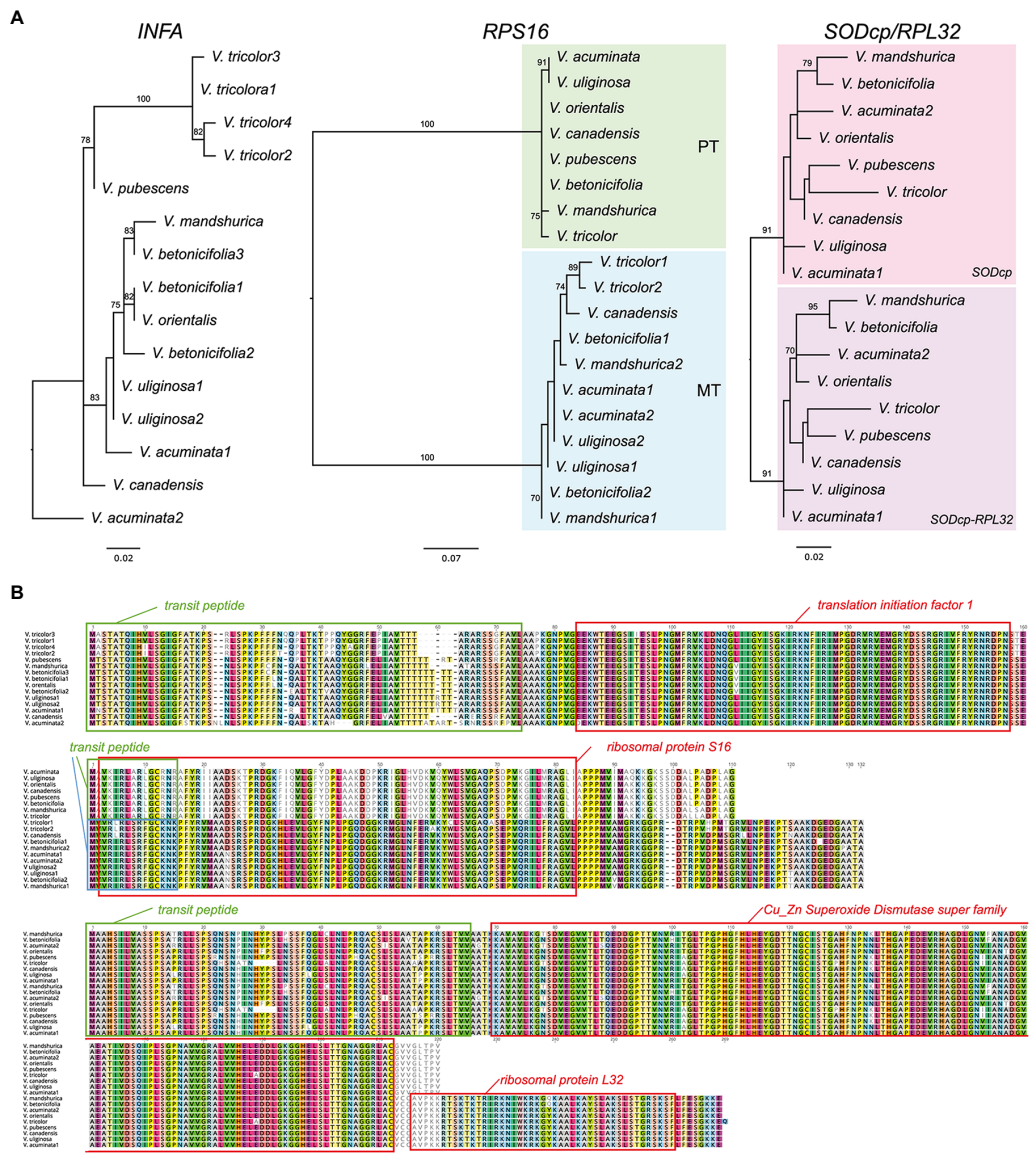
Nuclear-encoded *INFA*, *RPS16*, and *SODcp-RPL32* genes in *Viola*. **(A)** Phylogenetic analysis of the nuclear-encoded genes from *Viola*. The numbers after each species indicate the paralogs of each nuclear gene. **(B)** Amino acid sequence alignment of the nuclear encoded *INFA*, *RPS16*, and *SODcp-RPL32* copies from *Viola*. Boxes indicate transit peptide (green and blue) and conserved domains.

Seven additional *Viola* transcriptomes were surveyed for the identification of three nuclear-encoded plastid-targeted genes and provided clear evidence for potential functional replacement of the plastid-encoded *infA*, *rpl32*, and *rps16* to the nucleus. Interestingly, some *Viola* transcriptomes contain two or multiple copies. For example, *V. acuminate* transcriptomes contain two divergent *INFA*, *RPS16* (for mitochondrial), and *SODcp-RPL32* copies (Figure 3). *Viola betonicifolia* transcriptome contains three and two divergent copies of *INFA* and *RPS16* (for mitochondrial), respectively. *Viola tricolor* transcriptome contains four and two divergent copies of *INFA* and *RPS16* (for mitochondrial), respectively. *Viola uliginosa* transcriptome contains two divergent *INFA* and *RPS16* (for mitochondrial) copies. *Viola mandshurica* transcriptome contains two divergent *RPS16* (mitochondrial).

### Functional Transfer of Plastid Genes to the Nucleus in the Order Malpighiales

**Figure 4:**
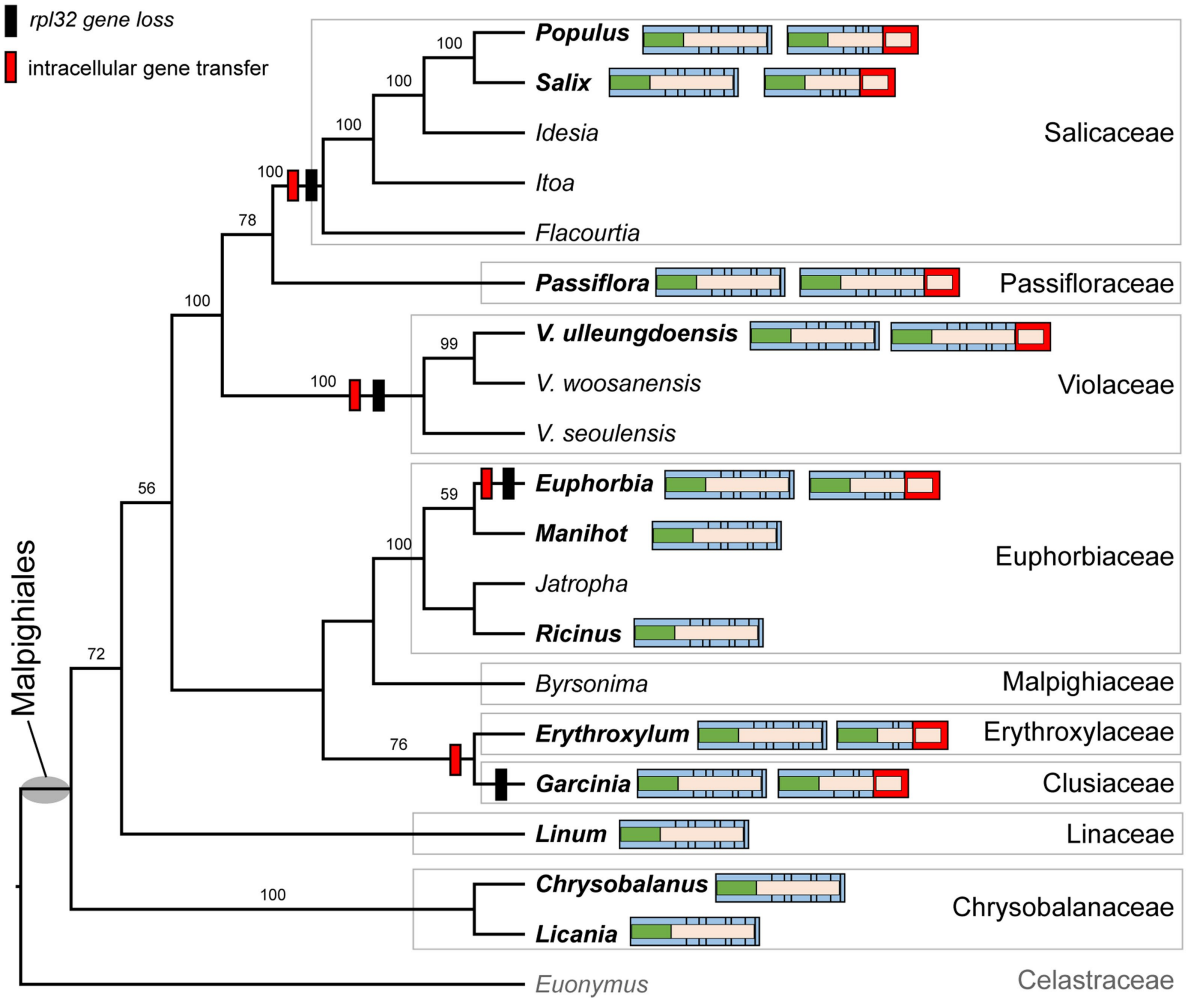
Phylogenetic distribution of nuclear-encoded *SODcp-RPL32* in Malpighiales. Phylogenetic relationships were inferred from 19 representative species in nine families of Malpighiales using an outgroup, *Euonymus* (Celastraceae). Bootstrap values based on 1,000 replicates and >50% are shown for each node. Schematic diagrams of the variable structure of the nuclear-encoded *SODcp* and *SODcp-RPL32* genes among selected Malpighiales are shown for 12 taxa in bold. Colors correspond to transit peptides, exons, and conserved domains (see Figure 2).

We determined the phylogenetic distribution of the plastid-encoded *infA*, *rpl32*, and *rps16* genes in 19 representative species from nine families of the order Malpighiales with *Euonymus japonicus* (Celastraceae, Order Celastrales) as an outgroup (Figure 4 and Supplementary Figure 5A). Our analysis showed that loss of the *infA* gene occurred in the common ancestor of Malpighiales, whereas loss of the *rpl32* and *rps16* genes was lineage-specific within Malpighiales (Figure 4 and Supplementary Figure 5A).

To investigate functional transfer to the nucleus, we queried the available transcriptome data from 11 Malpighiales species with the *Viola INFA*, *SODcp-RPL32*, and *RPS16* sequences (Supplementary Table 4). Except for *Garcinia* and *Ricinus*, transcriptomes containing ORFs with the conserved domain of “translation initiation factor A” domain were identified at the C-termini (Supplementary Figure 5B). The extended region of *Viola* was highly divergent with very low sequence identity (13.7–53.2%) to other Malpighiales (Supplementary Figure 5B). Two genera, *Euphorbia* and *Manihot*, contained two versions of the nuclear-encoded *INFA* gene (Supplementary Figure 5B). With the exception of *Manihot* and *Ricinus*, two transcripts with the conserved domain of “ribosomal protein S16” identified at the N-terminus were detected in the transcriptome (Supplementary Figure 5C). Phylogenetic analyses of the *RPS16* sequences from 11 Malpighiales species and both nuclear-encoded *RPS16* (for plastids and mitochondria) of *Viola* indicated two different origins for the transcripts (Supplementary Figure 5C). In the case of *rpl32*, nuclear *SODcp-RPL32* gene fusion was also identified in *Populus*, *Salix*, *Euphorbia*, *Erythroxylum*, *Passiflora*, and *Garcinia* transcriptomes (Figure 4). Amino acid sequence alignments of the chimeric gene products revealed structural degeneration or possible alternative mRNA splicing (Figure 3 and Supplementary Figure 6). Although the *Erythroxylum* plastome contained an intact *rpl32* gene, we found evidence for nuclear *SODcp-RPL32* gene fusion, suggesting the occurrence of an IGT event involving a common ancestor of the two families Erythroxylaceae and Clusiaceae (Figure 4).

### Distribution of *rpl32* and *rps16* Gene Loss Among Species of *Viola* in Korea

**Figure 5:**
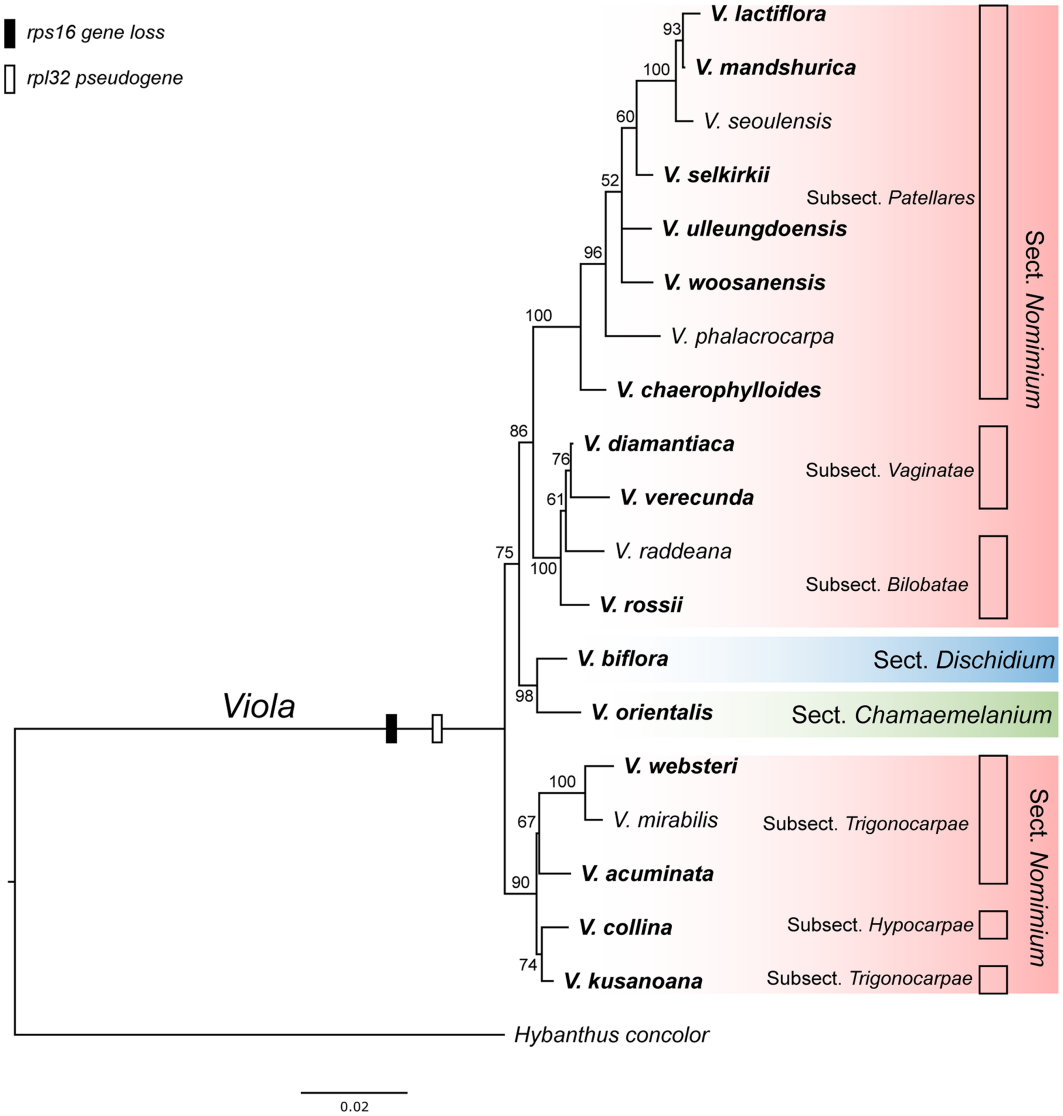
Phylogenetic distribution of plastid-encoded *rpl32* and *rps16* gene loss events in 19 representative species of the family Violaceae. The bootstrap value based on 1,000 replicates and >50% are shown for each node.

As the presence of the plastid-encoded *rpl32* and *rps16* genes in the plastomes varied among representative lineages of the order Malpighiales, we further surveyed to confirm whether gene loss events are species- or genus-specific among representative species of *Viola* in Korea. Thus, we surveyed 19 species of *Viola*, including sections of *Dischidium*, *Chamaemelanium*, and *Nomimium*, and amplified two IGS regions of *ndhF-trnL* and *trnK-trnQ via* PCR, given the locations of *rpl32* and *rps16*, respectively. The amplicon sizes of *ndhF-trnL* IGS ranged from 862 bp in *V. lactiflora* to 1,803 bp in *V. veracunda*. A BLAST search showed that the 19 examined species contained partial sequences of the *rpl32* gene with premature stop codons (Supplementary Figure 7). The amplicon sizes of the *trnK-trnQ* IGS ranged from 659 bp in *V. biflora* to 761 bp in *V. rossii*. BLAST searches revealed that the examined 19 species lacked detectable *rps16*-like sequences (Supplementary Figure 7). The phylogenetic distribution of losses involving the two genes suggested that the IGT event occurred in the most recent common ancestor of the *Viola* lineage in Korea (Figure 5).

## Discussion

In this study, we generated two complete plastomes of *V. ulleungdoensis* and *V. woosanensis* in the family Violaceae and explored the loss of three plastid-encoded genes, *infA*, *rpl32*, and *rps16*. Various analyses confirmed the transfer of these three plastid genes to the nuclear compartment in the genus *Viola* and in the order Malpighiales, further highlighting the dynamic process of IGT in angiosperms.

Sequenced plastomes of photosynthetic angiosperms generally contain 79 protein-coding genes, but the evolutionary timing of plastid gene loss events in multiple lineages has only been inferred (Jansen et al., 2007). These findings suggest that plastid gene transfer to the nucleus is still an ongoing process in angiosperms. However, successful functional gene transfer requires several steps because of the different nuclear machinery required for transcription and translation (Woodson and Chory, 2008). For example, a promoter, poly-A tail, and targeting sequence must be acquired after the integration of a plastid gene into the nuclear genome, and the subsequent pseudogenization and elimination of the original plastid gene sequence from its plastome. Transit peptides are critical for the transport of proteins into plastids in the cytosol. Three mechanisms for the acquisition of a targeting sequence in the nuclear genome have been reported (Ueda and Kadowaki, 2012); the acquisition of a novel transit peptide, acquisition of an existing transit peptide from a nuclear-encoded gene for plastids, and no acquisition due to the presence of an inherent transit peptide encoded in the gene. We determined that the *Viola* species examined in this study utilized all three strategies for the acquisition of transit peptides. For example, the transferred *infA* acquired a novel transit peptide based on the very low sequence identity between these proteins and no successful BLAST search results. In contrast, the transferred *rpl32* locus acquired an existing transit peptide by inserting into the nuclear-encoded plastid-targeted *SOD* gene as one exon. This second mechanism is known to occur uniquely in Malpighiale lineages (Cusack and Wolfe, 2007), whereas transfer of the plastid *rpl32* gene within another lineage (e.g., Ranunculaceae) followed a similar strategy to that involving *infA*. The generic structure of the nuclear *SODcp-RPL32* gene and two types of transcripts from *Viola* species suggested that the nuclear gene mRNA transcript was alternatively spliced and translated in the cytosol for import of both protein products into plastids (Figure 2). Likewise, the mangrove (*Bruguiera gymnorrhiza*) contains a chimeric gene with an alternative mRNA splicing mechanism, whereas *Popular* undergoes double duplication and complete sub-functionalization, which contains the *rpl32* protein with a *SODcp*-derived amino terminus and *SODcp* protein in plastids (Cusack and Wolfe, 2007; Ueda et al., 2007). The third strategy for the acquisition of transit peptides involves the plastid-encoded *rps16* gene in *Viola* (Figure 2). The nuclear-encoded *RPS16* for plastids lacks an N-terminal extension because of the presence of an internal targeting signal in the gene (Ueda et al., 2008). The transit peptide of *RPS16* is derived from a duplicate copy of a nuclear homolog, which is targeted to the mitochondrion (Ueda et al., 2008).

The analyzed *Viola* species contained two or multiple versions of the nuclear-encoded plastid-targeted genes, showing patterns of asymmetrical gene duplication. These patterns suggested that the duplicated genes independently originated at the different time within the *Viola* or that stochastic loss after ancient gene duplication in the genus. Allopolyploidy has played an important role in the speciation of the genus *Viola* (Ballard, 1996; Marcussen et al., 2012, 2014). This evolutionary history of *Viola* suggested that the duplication of the nuclear-encoded plastid-targeted genes may occurs *via* whole genome duplication or polyploidy after their functional replacement to the nucleus. The study of IGT can help to the detection of ancient polyploidization (Park et al., 2020). To better understand the important role of polyploidy in the evolutionary history of *Viola*, it will be necessary to sequence additional transcriptomes from *Viola* species spanning all recognized sections and subsections.

As the presence/absence of the *rpl32* and *rps16* genes varied within the order Malpighiales, we further sequenced each IGS region that included genes from 19 congeneric species of *Viola*. Our results indicated that the *rpl32* gene was pseudogenized, whereas the *rps16* gene was completely lost from the plastome (Supplementary Figure 7). These results suggested that IGT involving the two genes must have occurred in the most-recent common ancestor of violet species in Korea, that is, most likely genus-specific; these became pseudogenes or were completely lost upon their transfer to the nuclear compartment.

Although nine plastid gene losses (*infA*, *rpl20*, *rpl22*, *rpl32*, *rps7*, *rps16*, *rps19*, *ycf1*, and *ycf2*) have been documented in Malpighiales (Menezes et al., 2018; Shrestha et al., 2020), molecular characterization of these events across the order has been limited. Of these gene loss events, we focused specifically on the fate of the plastid-encoded *infA*, *rpl32*, and *rps16* loci in Malpighiales to better understand the dynamics of IGT. As plastid phylogenomic relationships of Malpighiales from nine families were reconstructed, we demonstrated that the *infA* gene loss occurred in the common ancestor of the order Malpighiales, whereas *rpl32* and *rps16* were lost independently multiple times within the order. Transcriptome data provided evidence for successful lineage- or species-specific IGT (Figure 4 and Supplementary Figures 5, 6). Plastid-encoded *infA* underwent multiple independent transfers to the nucleus (Millen et al., 2001). Our findings suggested a single transfer of the plastid *infA* to the nucleus in the most-recent common ancestor of the order Malpighiales although we did not find evidence for *Garcinia* and *Ricinus*. The transcriptomes of three genera, *Manihot*, *Euphorbia*, and *Populus*, contained two versions of the nuclear-encoded *INFA*, which may be associated with whole genome or segmental gene duplication. However, the phylogenetic placement of nuclear-encoded *INFA* suggests different origins for the duplicated copies. For example, *Manihot* and *Euphorbia* copies were duplicated by a species-specific event involving nuclear-encoded *INFA* (Supplementary Figure 6). In the case of *Populus*, *INFA* was duplicated in the common ancestor of *Populus* and *Salix*, and one copy loss occurred in *Salix*, resulting in the presence of two copies in *Populus*. The nuclear-encoded *SODcp-RPL32* is a well-characterized example of gene transfer in Malpighiales (Cusack and Wolfe, 2007), in which the plastid *rpl32* is integrated into an intron of the nuclear *SOD* gene in the Malpighiales ancestor. Comparative analyses of the nuclear-encoded *SODcp-RPL32* in Malpighiales revealed that the transfer events were lineage-specific (Figure 4) and argued against the scenario of a single ancestral event. However, further studies, including more Malpighiales transcriptomes, are needed to confirm whether the formation of the *SODcp-RPL32* chimeric gene is a general or specific phenomenon. Inactivation of plastid genes is favored after nuclear gene activation (Martin and Herrmann, 1998), and intermediate stages of the gene transfer process have been identified in Ranunculaceae (Park et al., 2020). We found co-existence of intact and transcribed gene copies in the *Erythroxylum* nucleus and plastids, suggesting that the IGT event occurred in the common ancestor of *Erythroxylum* and *Garcinia*. Likewise, *Passiflora* contains both copies of the nuclear-encoded *SODcp-RPL32* and plastid *rpl32* copies. A recent study also identified the *SODcp-RPL32* chimeric gene in many *Passiflora* species that lack the *rpl32* in their plastomes and in some *Passiflora* groups that contain plastid *rpl32* (Shrestha et al., 2020). These results indicated that putative functional transfer of plastid *rpl32* to the nucleus occurs in the common ancestor of the genus *Passiflora*. The nuclear-encoded plastid *RPS16* is a good example of gene substitution for functional replacement involving the nucleus. It has been replaced by the nuclear-encoded mitochondrial *RPS16*, which had already been transferred to the nucleus *via* IGT (Keller et al., 2017). The transit peptides of the nuclear-encoded *RPS16* contain a dual targeting signal for mitochondria and plastids, which is an internal signal without an N-terminal extension (Ueda et al., 2008). Among Malpighiales, plastid *rps16* was lost independently at least seven times in our samples, and an expanded survey detected two versions of the nuclear-encoded *RPS16* copies that were from two different origins (Supplementary Figure 5C). Although *Ricinus* and *Garcinia* plastomes contain the plastid *rps16* gene, we found nuclear-encoded plastid *RPS16* copies (Supplementary Figure 5C). These results suggested that gene substitution involving plastids occurred in the most-recent common ancestor of Malpighiales, and this may have facilitated the loss of the plastid-encoded *rps16* gene from its plastomes. This finding also indicated that gene substitution of plastid-encoded *rps16* was in the intermediate stage within Malpighiales.

## Data Availability Statement

The datasets presented in this study can be found in online repositories. The names of the repository/repositories and accession number(s) can be found at: https://www.ncbi.nlm.nih.gov/genbank/, MK228834 and https://www.ncbi.nlm.nih.gov/genbank/, MK228835.

## Author Contributions

JY, SP, S-CK, and J-HP conceived and designed experiments. JY generated and analyzed the data and wrote a draft of the manuscript, which SP edited and S-CK revised. SP analyzed transcriptome results. H-YG collected samples and conducted PCR and sequencing procedures. All authors contributed to the article and approved the submitted version.

## Conflict of Interest

The authors declare that the research was conducted in the absence of any commercial or financial relationships that could be construed as a potential conflict of interest.

## Publisher’s Note

All claims expressed in this article are solely those of the authors and do not necessarily represent those of their affiliated organizations, or those of the publisher, the editors and the reviewers. Any product that may be evaluated in this article, or claim that may be made by its manufacturer, is not guaranteed or endorsed by the publisher.

## Footnotes

^1^ www.geneious.com

^2^ https://chlorobox.mpimp-golm.mpg.de/OGDraw.html

^3^ https://sites.google.com/a/ualberta.ca/onekp/

^4^ https://phytozome.jgi.doe.gov/pz/portal.html
