# Supplementary material for: Characterization and Dynamics of Intracellular Gene Transfer in Plastid Genomes of Viola (Violaceae) and Order Malpighiales
Source: Front Plant Sci. 2021 Aug 27;12:678580. doi: 10.3389/fpls.2021.678580 (PMC8429499; doi:10.3389/fpls.2021.678580)
Supplement: Supplementary file 1 [file Presentation_1.pdf]

Article title: Characterization and dynamics of intracellular gene transfer in plastid genomes of *Viola* (Violaceae) and order Malpighiales

Authors: JiYoung Yang, Seongjun Park, Hee-Young Gil, Jae-Hong Pak, Seung-Chul Kim

Supplementary information

Figure S1. Structural alignments of seven Violaceae plastomes using Mauve.
Figure S2. Domain structure and amino acid sequences of nuclear-encoded *INFA* from *V. acuminata*.
Figure S3. Domain structure and amino acid sequences of nuclear-encoded *SODcp-RPL32* from *V. acuminata*.
Figure 4S. Domain structure and amino acid sequences of nuclear-encoded *RPS16* from *V. acuminata*.
Figure 5S. Plastid *infA* and *rps16* gene loss event in Malpighiales.
Figure 6S. Amino acid alignment and gene/domain structure of nuclear-encoded *SODcp-RPL32*.
Figure 7S. Nucleotide alignment of the plastid *rpl32* and *rps16* genes for Violaceae

Table S1. Primers information used for amplification.
Table S2. The accession number for phylogenetic analysis of Malpighiales.
Table S3. The accession number for phylogenetic analysis of Violaceae.
Table S4. Information of nuclear-encoded *INFA*, *SODcp-RPL32* and *RPS16* genes.

Supplementary Figure 1. Structural alignments of seven Violaceae plastomes using Mauve.

The colored block represents collinear sequence blocks shared by all plastomes. The height of each bar reflects sequence similarity. Individual genes and strandedness are represented below the *Viola ylleungdoensis* genome block. Only one copy of the inverted repeat (IR) is shown for each plastome and pink boxes below each plastome block indicate its IR. Gray boxes with alignment of each region show an loss events that are shared by all plastomes.

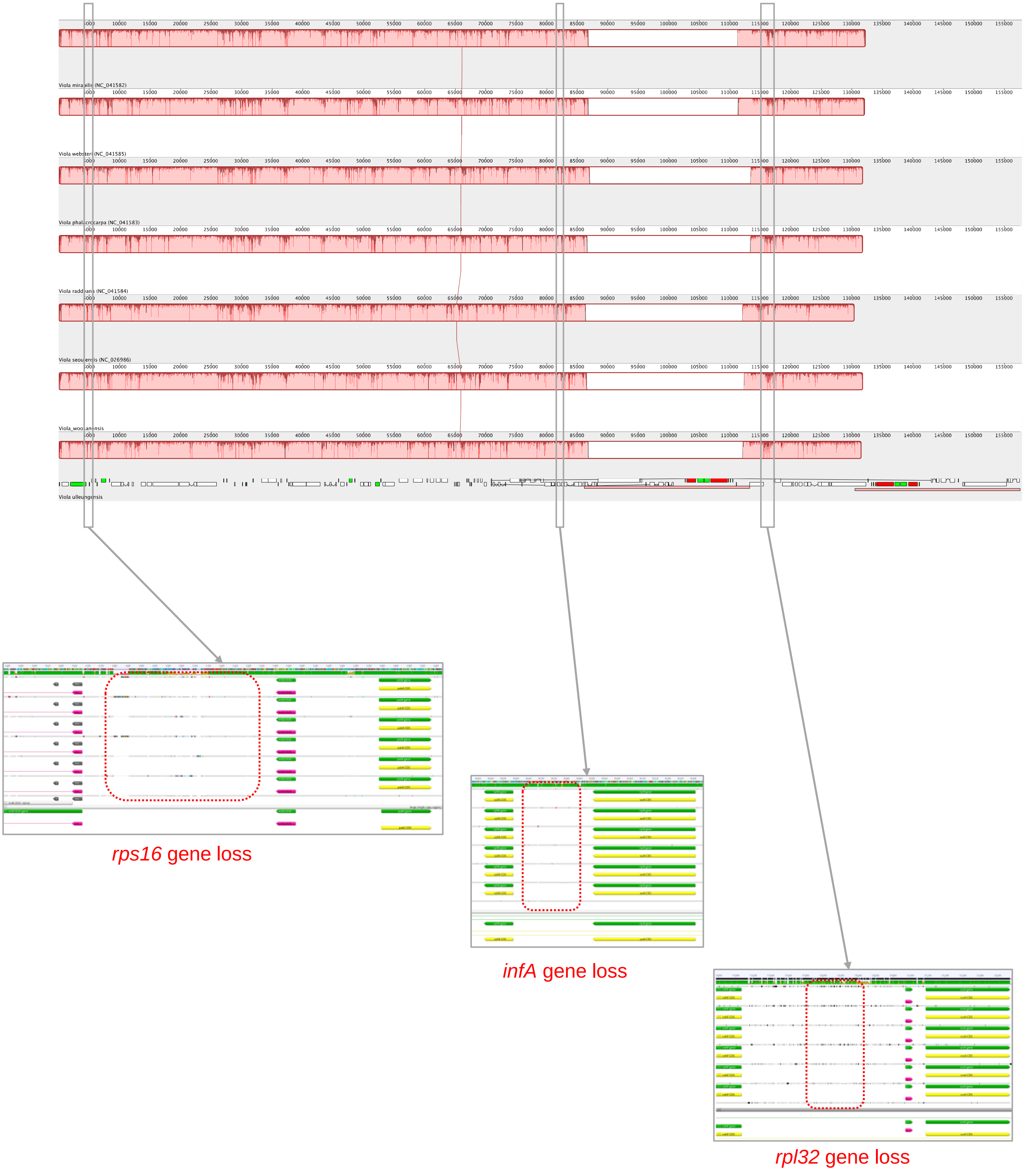

Supplementary Figure 2. Domain structure and amino acid sequences of the nuclear-encoded *INFA* from *V. acuminata*.

Boxes indicate chloroplast transit peptide (pink) and a conserved domain of translation initiation factor 1 (red). Schematic of the domain structure of *infA* as identified by the CDD at the NCBI.

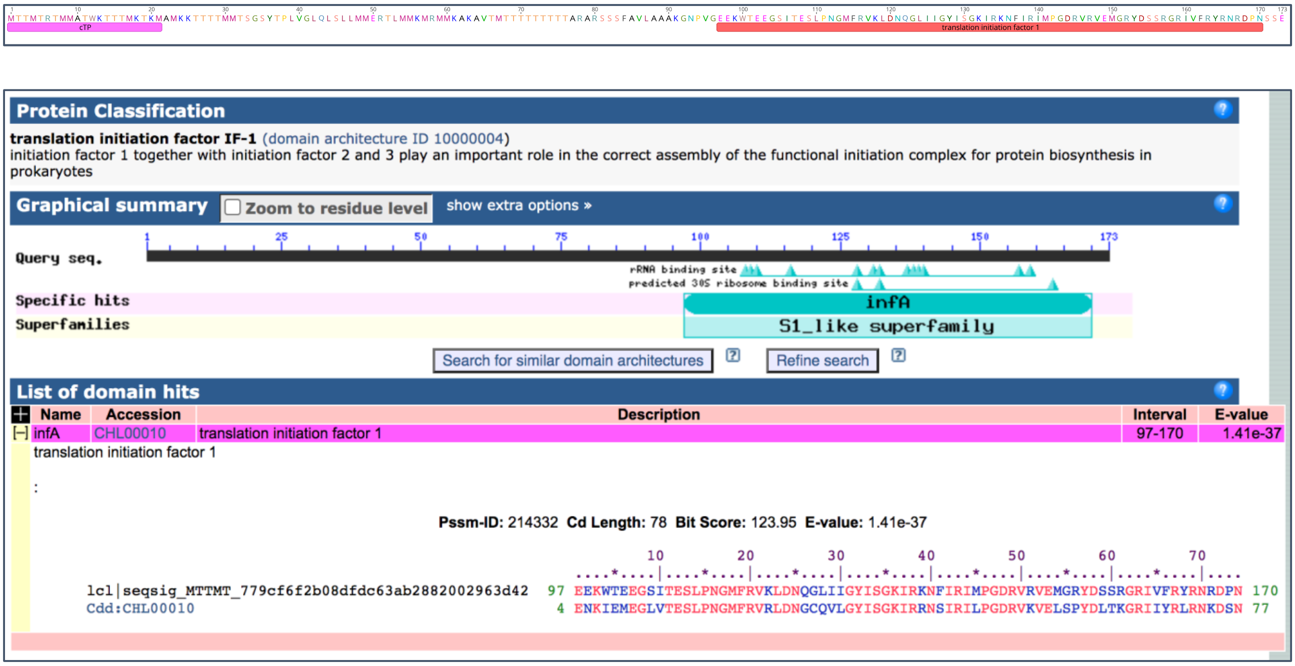

frontiers

Supplementary Figure 3. Domain structure and amino acid sequences of the nuclear-encoded *SODcp-RPL32* from *V. acuminata*.

Boxes indicate chloroplast transit peptide (pink) and a conserved domain of Cu-Zu Superoxide Dismutase super family (red). Schematic of the domain structure of *SODcp-RPL32* as identified by the CDD at the NCBI. A. The nuclear-encoded SODcp-RPL32 gene. B. The nuclear-encoded SODcp gene.

A

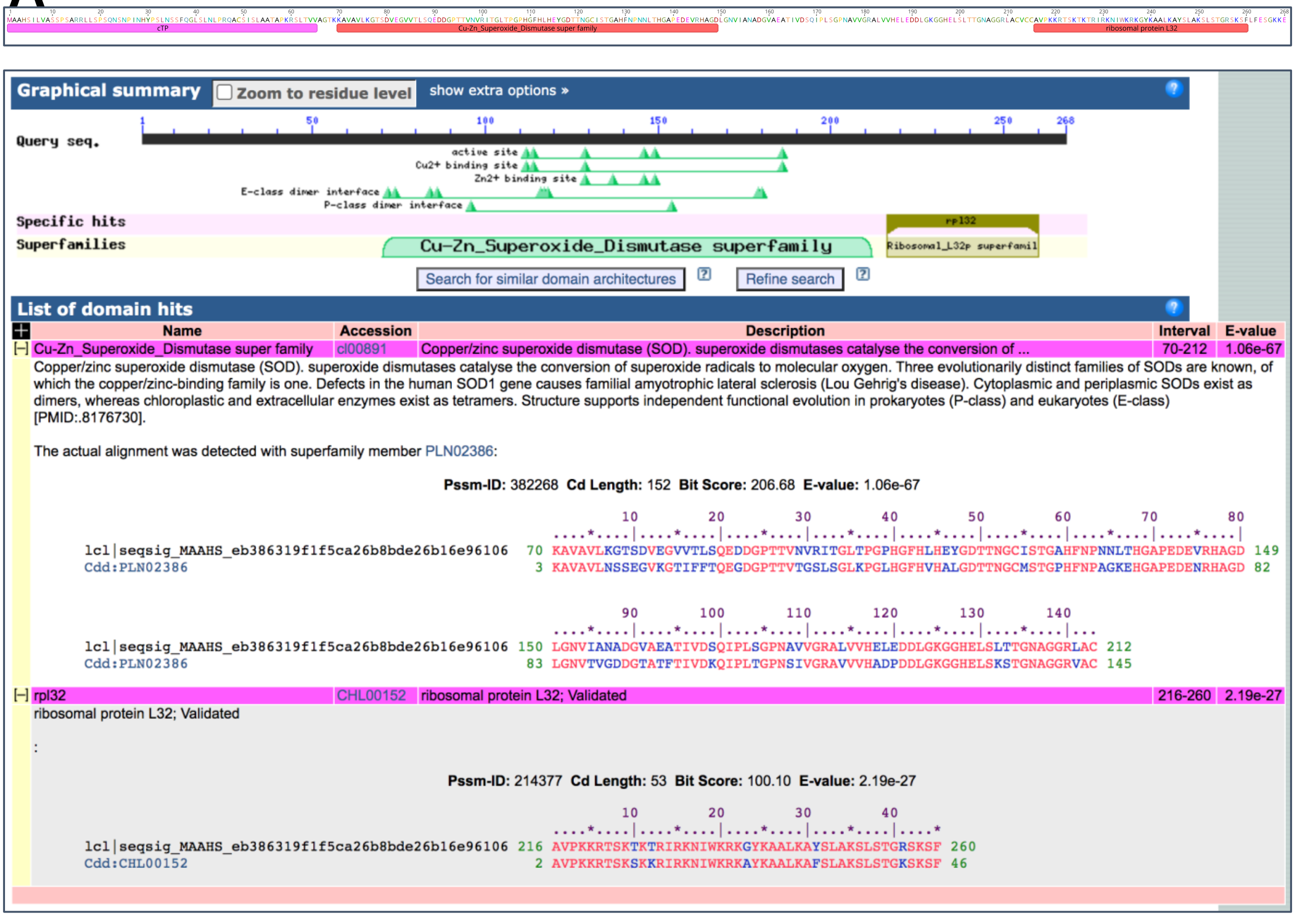

B

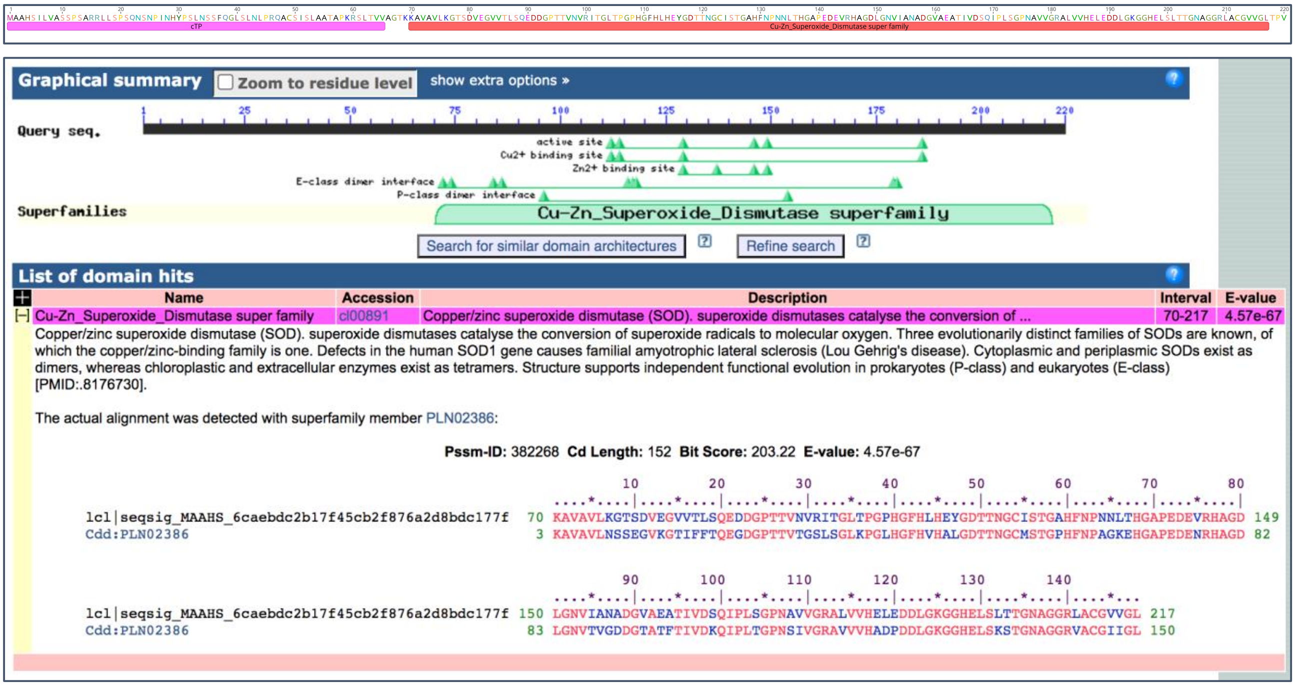

Supplementary Figure 4. Domain structure and amino acid sequences of the nuclear-encoded *RPS16* from *V. acuminata*.

Boxes indicate chloroplast transit peptide (pink) and a conserved domain of ribosomal protein S16 (red). Schematic of the domain structure of *RPS16* as identified by the CDD at the NCBI. A. The nuclear-encoded plastid-targeted *RPS16* gene. B. The nuclear-encoded mitochondrial-targeted *RPS16* gene.

A

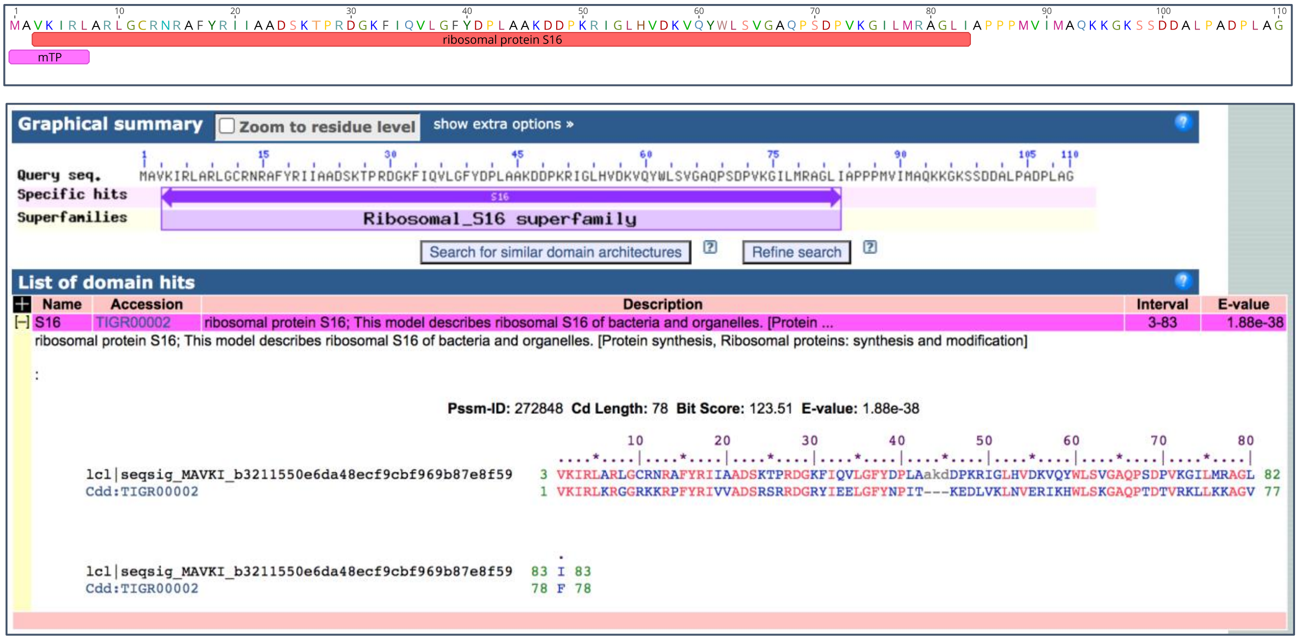

B

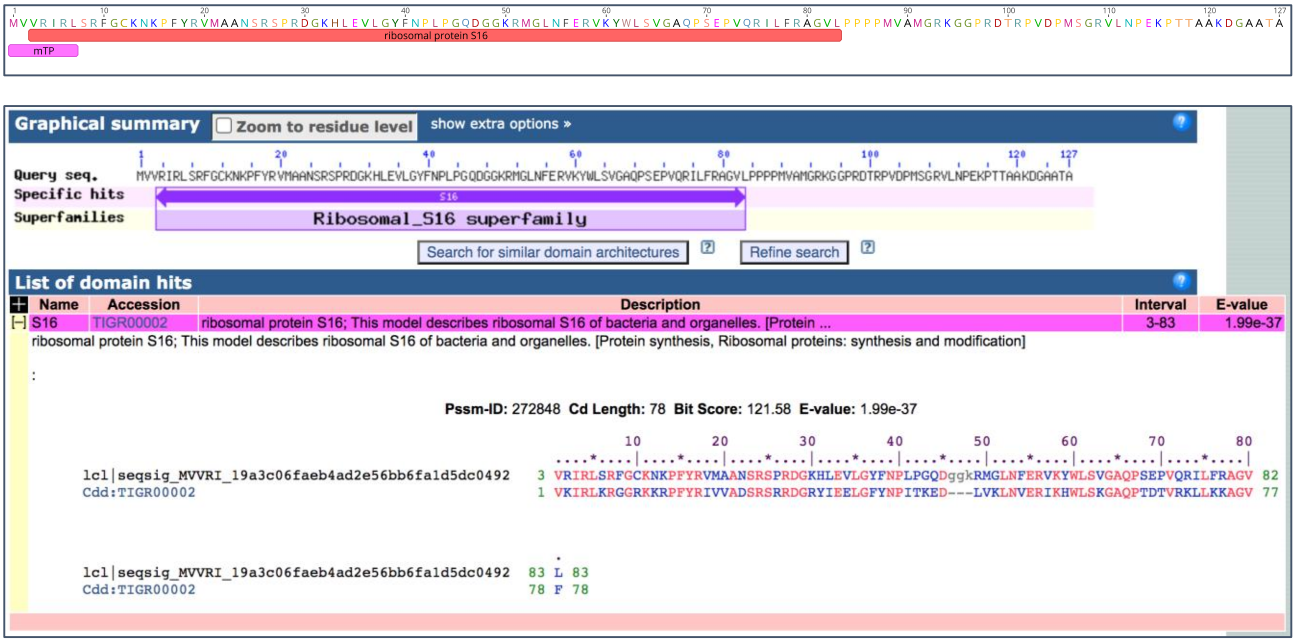

Supplementary Figure 5. Plastid *infA* and *rps16* gene loss event in Malpighiales.

A. Phylogenetic relationships inferred from 19 representative phylogenetic relationships of the order Malpighiales with an outgroup. The bootstrap value based on 1,000 replicates and > 50% is shown for each node. B. Amino acid alignment and gene/domain structure of the nuclear-encoded *INFA*. C. Amino acid alignment and gene/domain structure of the nuclear-encoded *SODcp-RPL32*. Red boxes indicate a conserved domain of each protein.

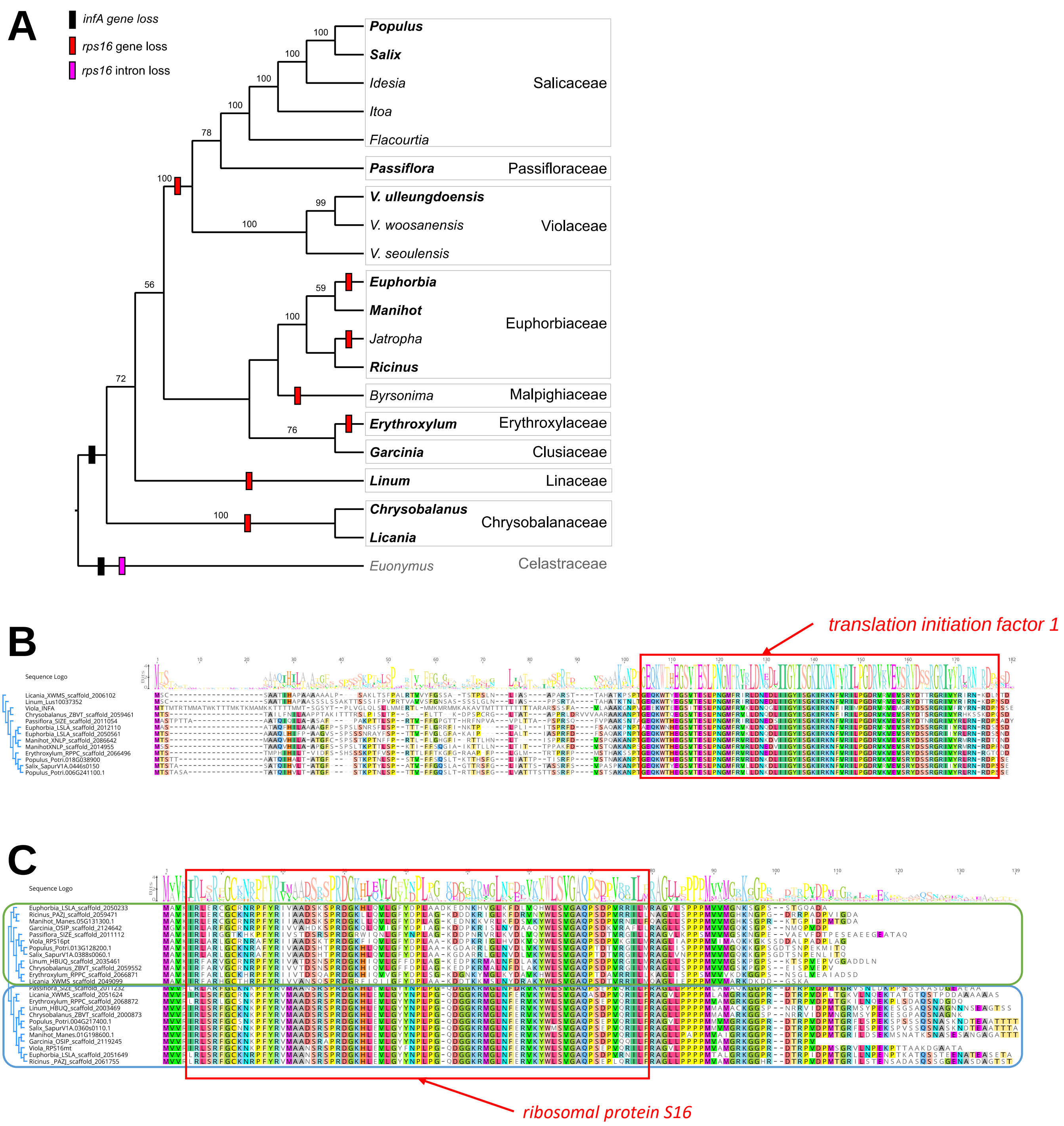

Supplementary Figure 6. Amino acid alignment and gene/domain structure of nuclear-encoded *SODcp-RPL32*.

A

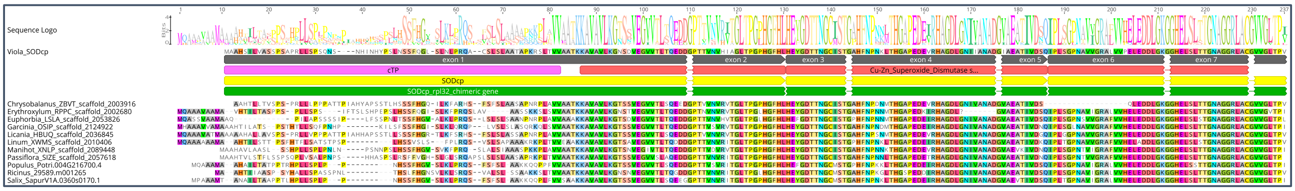

B

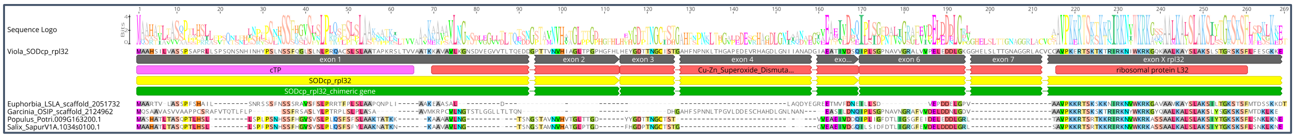

Supplementary Figure 7. Nucleotide alignment of the plastid *rpl32* and *rps16* genes for Violaceae.

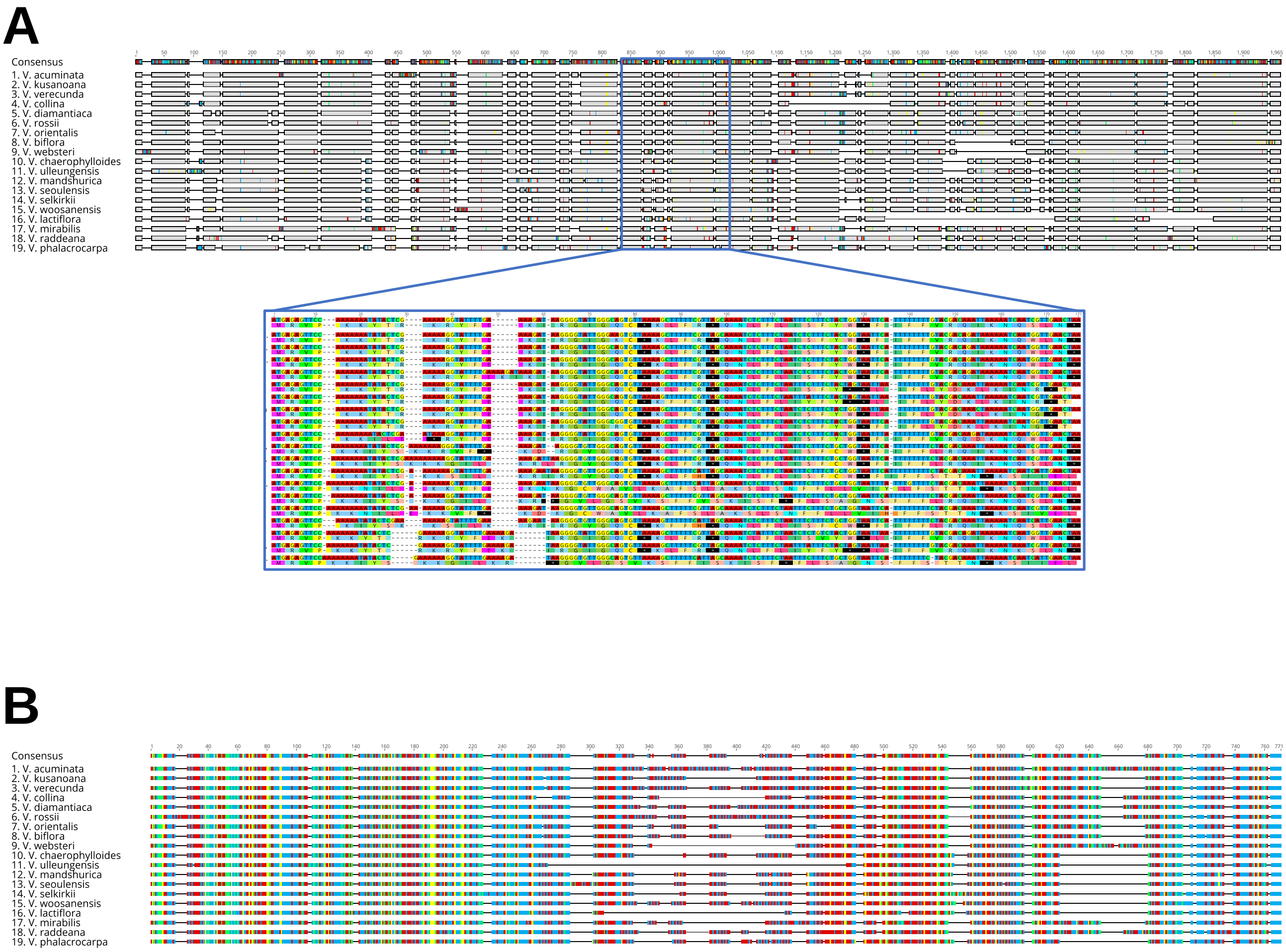

Table S1. Primers information used for amplification.

	Primer	Sequence (5’→ 3’)
Nuclear encoded *rpl32* gene	25F	CATTATCCTCAGACATCAGCTGCA
	870R	CTGCTTCTACTCCTTTTTCCCACT
Chloroplast encoded *rpl32* gene	2163F	CCGGAAAAATTATCAGCCCCATTC
	4067R	AAATTCAAAGATGTTTATGCCGCCA
	2671F	AGGAATGATAAAAAGACATGCCGA
	3416F	AAAAATCCCCACCCTGCAAATTAT
Nuclear encoded *rps16* gene	61F	TGTGCGAGTATGGCAGTGAAGATT
	442R	TCAGTGTTTCATGGCAATTGACTT
Chloroplast encoded *rps16* gene	489F	TGACGAAGAACCAATACCAATAGT
	1288R	AAAAGCCGAGTACTCTACCATTGA

Table S2. The accession number for phylogenetic analysis of Malpighiales.

**Family**	**Species**	**Accession number**
**Salicaceae**	*Populus alba*	NC008235
	Salix purpurea	NC026722
	Idesia polycarpla	NC032060
	Itoa orientalis	NC037411
	Flacourtia indica	NC037410
	Passiflora edulis	NC034285
**Euphorbiaceae**	*Manihot esculenta*	NC010433
	Ricinus communis	NC016736
	Euphorbia hainanensis	MH049548
	Jatropha curcas	NC012224
**Erythroxylaceae**	*Erythroxylum novogranatense*	NC030601
**Clusiaceae**	*Garcinia mangostana*	NC036341
**Malpighiaceae**	*Brysonima coccolobifolia*	NC013791
	Brysonima crassifolia	NC013792
**Chrysobalanaceae**	*Chrysobalanus icaco*	NC024061
	Hirtella racemosa	NC024060
	Licania sprucei	NC024065
**Linaceae**	*Linum usitatissimum*	NC036356
**Celastraceae**	*Euonymus japonicus*	NC028067

Table S3. The accession number for phylogenetic analysis of Violaceae.

	atpF-H intergenic region	*psbA-trnH* intergenic region	*rpl16* intron	*trnL-F* intergenic region
Viola acuminata	GQ262554.1	GQ262597.1	GQ262683.1	DQ085890.1
V. biflora	GQ262549.1	GQ262592.1	GQ262678.1	DQ085922.1
V. chaerophylloides	GQ262570.1	GQ262613.1	GQ262699.1	DQ085902.1
V. collina	GQ262552.1	GQ262595.1	GQ262681.1	DQ085887.1
V. diamantica	GQ262568.1	GQ262611.1	GQ262697.1	DQ085900.1
V. kusanoana	GQ262558.1	GQ262601.1	GQ262687.1	DQ085893.1
V. mandshurica	GQ262580.1	GQ262623.1	GQ262709.1	DQ085913.1
V. orientalis	GQ262550.1	GQ262593.1	GQ262679.1	DQ085929.1
V. rossii	GQ262566.1	GQ262609.1	GQ262695.1	DQ085898.1
V. selkirkii	GQ262585.1	Q262628.1	GQ262714.1	DQ085920.1
V. seoulensis	GQ262581.1	GQ262624.1	Q262712.1	DQ085914.1
V. verecunda	GQ262563.1	GQ262606.1	GQ262692.1	DQ085896.1
V. websteri	GQ262556.1	GQ262599.1	GQ262685.1	DQ085892.1
Hybanthus concolor	GQ262591.1	GQ262634.1	GQ262720.1	GQ262538.1

Table S4. Information of nuclear-encoded *INFA*, *SODcp-RPL32* and *RPS16* genes

Order	Famliy	Species	*INFA*	*SODcp*	*SODcp-RPL32*	*RPS16*
Malpighiales	Salicaceae	*Populus trichocarpa*	Potri.006G241100, Potri.018G038900	Potri.004G216700.4	Potri.009G163200	Potri.004G217400.1, Potri.013G128200.1
		Salix purpurea	SapurV1A.0446s0150	SapurV1A.0360s0170.1	SapurV1A.1034s0100	SapurV1A.0360s0110.1, SapurV1A.0388s0060.1
	Passifloraceae	*Passiflora caerulea*	SIZE_scaffold_2011054	SIZE_scaffold_2057618	-	SIZE_scaffold_2011112, SIZE_scaffold_2011232
	Violaceae	*Viola ulleungdoensis*	MZ379779	MW716506	MW716506	MW716505
	Euphorbiaceae	*Euphorbia mesebyranthemum*	LSLA_scaffold_2012110, LSLA_scaffold_2050561	LSLA_scaffold_2053826	LSLA_scaffold_2051732	LSLA_scaffold_2050233, LSLA_scaffold_2051649
		Manihot grahamii	XNLP_scaffold_2014955, XNLP_scaffold_2086642	XNLP_scaffold_2089448	x	Manes.05G131300.1, Manes.01G198600.1
		Ricinus communis	x	29589.m001265	x	29747.m001059, 27395.m000079
	Erythroxylaceae	*Erythroxylum coca*	RPPC_scaffold_2066496	RPPC_scaffold_2002680	RPPC_scaffold_2068218	RPPC_scaffold_2066871, RPPC_scaffold_2066872
	Clusiaceae	*Garcinia livingstonei*	x	OSIP_scaffold_2124922	OSIP_scaffold_2124962	OSIP_scaffold_2124642
	Linaceae	*Linum perenne*	XWMS_scaffold_2006102	XWMS_scaffold_2010406	x	XWMS_scaffold_2051624 , XWMS_scaffold_2049099
	Chrysobalanacae	*Chrysobalanus icaco*	ZBVT_scaffold_2059461	ZBVT_scaffold_2003916	x	ZBVT_scaffold_2059552, ZBVT_scaffold_2000873
		Licania michauxii	Lus10037352a, Lus10035773a	HBUQ_scaffold_2036845	x	HBUQ_scaffold_2035461, HBUQ_scaffold_2003469
